# Serial visual reversal learning in captive black-handed spider monkeys, *Ateles geoffroyi*

**DOI:** 10.1007/s10071-024-01897-z

**Published:** 2024-08-13

**Authors:** Jules Dorschner, Laura Teresa Hernandez Salazar, Matthias Laska

**Affiliations:** 1 IFM Biology, Linköping University, Linköping, SE-581 83 Sweden; 2 Instituto de Neuroetologia, Universidad Veracruzana, Xalapa, C.P. 91000 Mexico

**Keywords:** Serial visual reversal learning, Cognition, Black-handed spider monkey, *Ateles geoffroyi*

## Abstract

Recent research suggests that socio-ecological factors such as dietary specialization and social complexity may be drivers of advanced cognitive skills among primates. Therefore, we assessed the ability of 12 black-handed spider monkeys (*Ateles geoffroyi*), a highly frugivorous platyrrhine primate with strong fission-fusion dynamics, to succeed in a serial visual reversal learning task. Using a two-alternative choice paradigm we first trained the animals to reliably choose a rewarded visual stimulus over a non-rewarded one. Upon reaching a pre-set learning criterion we then switched the reward values of the two stimuli and assessed if and how quickly the animals learned to reverse their choices, again to a pre-set learning criterion. This stimulus reversal procedure was then continued for a total of 80 sessions of 10 trials each. We found that the spider monkeys quickly learned to reliably discriminate between two simultaneously presented visual stimuli, that they succeeded in a visual reversal learning task, and that they displayed an increase in learning speed across consecutive reversals, suggesting that they are capable of serial reversal learning-set formation with visual cues. The fastest-learning individual completed five reversals within the 80 sessions. The spider monkeys outperformed most other primate and nonprimate mammal species tested so far on this type of cognitive task, including chimpanzees, with regard to their learning speed in both the initial learning task and in the first reversal task, suggesting a high degree of behavioral flexibility and inhibitory control. Our findings support the notion that socio-ecological factors such as dietary specialization and social complexity foster advanced cognitive skills in primates.

## Introduction

Comparative studies on the cognition of primates found marked differences between species in a variety of abilities including perception, learning, memory, decision making, problem solving, and tool use, to name but a few (Schwartz and Beran 2022). Such studies allow us to gain insight into the evolutionary selective pressures that may act upon a given species and which may underlie between-species differences in cognitive performance on a given task. Further, they allow us to test hypotheses as to the evolutionary origins of human cognition. Early studies on different types of learning tasks in nonhuman primates suggested a clear phylogenetic trend with great apes outperforming catarrhine primates which, in turn, were thought to perform better than platyrrhines, and strepsirrhines scoring at the bottom of such rankings (e.g. Harlow and Bromer 1939; Rumbaugh et al. 1996; Beran et al. 2014; Washburn and Walters 2022). However, more recent studies, including meta-analyses and Bayesian modelling of comparative data on cognitive performance, call this phylogenetic trend into question (Deaner et al. 2006; Reader et al. 2011). Instead, accumulating evidence now suggests that cognitive differences between primate species may be more domain- or task-specific rather than domain- or task-general (Johnson et al. 2002; Amici et al. 2010; Schmitt et al. 2012).

To this day, certain primate taxa have been studied much more intensively with regard to their cognitive abilities than others. This should not be surprising as the great apes, for example, have traditionally been of interest due to their close phylogenetic relationship to humans, and rhesus macaques, a catarrhine primate, have been widely used due to the large body of knowledge about their maintenance in captive settings. Platyrrhine primates, on the other hand, have been clearly understudied so far concerning their cognitive performance, perhaps with the exception of the capuchin monkey (Gossette and Inman 1966; Gossette and Gossette 1967; D’Amato and Salmon 1984; Visalberghi 1997; Beran et al. 2008).

The spider monkey (*Ateles geoffroyi*) is a highly frugivorous platyrrhine primate displaying strong fission-fusion dynamics (Gonzalez-Zamora et al. 2009; Campbell 2008). Both of these traits are thought to foster the evolution of advanced cognitive capabilities. The complex spatiotemporal distribution of fruit, for example, requires cognitive skills such as mental maps, long-term spatial and sensory memory, and decision-making skills in order to remember the location of patchily distributed food sources, to efficiently predict times and places to forage, and to optimize food selection (Milton 1981). Similarly, the complex social relationships in a multi-male/multi-female society and the temporary separation of a social group into smaller foraging units of variable composition require cognitive skills such as multimodal individual recognition of conspecifics and communication skills to keep track of changes in social relationships (Aureli et al. 2008).

The few studies on cognitive performance in spider monkeys to date indeed suggest that they generally outperform other platyrrhines on physical cognition tasks, whereas hardly anything is known about their relative performance in social cognition tasks (Reader et al. 2011). In certain tests of physical cognition such as patterned-string problems and numerical cognition tasks spider monkeys have been found to clearly score better than catarrhine and strepsirrhine primates and to perform at the same high level as great apes (Deaner et al. 2006; Bosshard et al. 2022). Therefore, we decided to assess serial visual reversal learning in a group of captive black-handed spider monkeys, a type of cognitive task that has not been employed with this species so far.

Stimulus reversal learning is a classical and widely used test of physical cognition. It is considered as a measure of behavioral flexibility and inhibitory control (Shettleworth 2010; Lea et al. 2020). Stimulus reversal learning is based on a two-choice paradigm in which an animal is first rewarded for choosing a certain one of two simultaneously presented stimuli, e.g. a black object rather than a white object, and then is supposed to learn to reverse its choice, that is, to choose the white object rather than the black one. In *serial* stimulus reversal learning this process of reversing the reward values of the two stimuli is repeated several times.

Comparative studies on both primates and non-primate vertebrates found that virtually all species tested so far needed more trials to reach the learning criterion in the first reversal condition compared to the initial learning. Possible explanations for this phenomenon include, but are not restricted to, perseverance, the development of a “win-stay, lost-shift” strategy, and a more associative rather than rule-based form of learning (Beran et al. 2008).

With successive reversals some species such as pigeons (*Columba livia*), rats (*Rattus norvegicus*), harbor seals (*Phoca vitulina*), and rhesus macaques (*Macaca mulatta*) have been shown to either display an increase in learning speed, that is, they need fewer and fewer trials to reach the learning criterion, or an increase in trial-2 performance with consecutive reversals, whereas other species such as painted turtles (*Chrysemys picta*) and the goldfish (*Carassius auratus*) failed to show such an increase in performance across reversals (Mackintosh et al. 1985; Erdsack et al. 2022). With a sufficiently high number of reversals, some species such as rats and chimpanzees even reach one-trial learning, meaning that they reliably choose the rewarded stimulus from the second trial onwards after the reward values of the two stimuli have been reversed (Schusterman 1964; Mackintosh et al. 1968).

Studies on serial stimulus reversal learning usually employ one of two possible approaches:

Studies of the first type employ a fixed number of trials per problem. (Please note that in this context, a “problem” is defined as a given pair of stimuli that are to be discriminated.) Here, the reward values of the rewarded stimulus and the non-rewarded stimulus are reversed after a given number of trials on a problem (often 6 or 10 trials), regardless of performance, that is, regardless of whether an animal has learned the reward values of the two stimuli or not. Accordingly, studies of this type allow for a high number of reversals, but only allow for assessing trial-2 performance, that is, whether an animal chooses the correct option in the second trial after a reversal has been implemented, as the only measure of performance (Beran et al. 2008; Rayburn-Reeves et al. 2017; Hassett and Hampton 2017; Kumpan et al. 2020).

Studies of the second type employ a performance criterion. Here, the reward values of the rewarded and the non-rewarded stimulus are not reversed until an animal has attained a given level of performance, that is, reached a pre-set learning criterion (e.g. 80% correct choices in a given number of consecutive trials) on a problem. Accordingly, studies of this type only allow for a low number of reversals but instead allow for assessing not only trial-2 performance as the measure of cognitive ability, but other, and more informative, measures such as the number of trials (or sessions) till criterion, and/or the total number of errors till criterion. Further, only with studies of the second type can an experimenter be sure that the animal has indeed learned that the reward values of the stimuli have been reversed (Joly et al. 2014; Jackson et al. 2019; Cantwell et al. 2022; Loyant et al. 2023). We therefore decided to employ the second approach in our study.

Given the paucity of data on cognitive performance in spider monkeys and the availability of comparative data on serial stimulus reversal learning from other primate and non-primate species, it was therefore the aim of the present study to assess

1. how quickly spider monkeys learn to reliably discriminate between two simultaneously presented visual stimuli,
2. how quickly they learn a first reversal of the reward values of the visual stimuli,
3. if the spider monkeys display an increase in learning speed across consecutive reversals,
4. if they reach one-trial learning in this type of cognitive task, and.
5. how the performance of the spider monkeys in this cognitive task compares to that of other species tested previously.

## Methods

### Animals

The study was carried out with 12 adult black-handed spider monkeys (*Ateles geoffroyi*). The group consisted of seven males and five females, aged between eight and twelve years. The animals were maintained at the field station UMA Doña Hilda Ávila de O’Farrill of the Universidad Veracruzana, located in a nature reserve near Catemaco, state of Veracruz, Mexico. They were housed in a roofed enclosure of 20 × 10 × 8 m which was subdivided into ten equally-sized compartments. The enclosures were connected by sliding doors which were usually kept open allowing the animals to interact with each other but could be closed for temporary separation of individuals. The animals were exposed to natural light and ambient temperature and provided with fresh fruits and vegetables once per day and occasionally with seeds and edible foliage to complement their diet. The enclosures were equipped with branches, ropes, tires, perches, sleeping boxes and further enrichment designed for climbing, swinging, and resting. The experiments were carried out in the morning before feeding and no food deprivation schedule was adopted. The animals had participated in previous studies on their cognitive abilities, e.g. in numerical cognition tasks (Bosshard et al. 2022) as well as in sensory discrimination tasks (Pereira et al. 2021) and were thus accustomed to participating in behavioral tests and to temporary separation. All animals were tested individually in order to prevent interference from, and distraction by, the other animals.

### Test apparatus

**Fig. 1:**
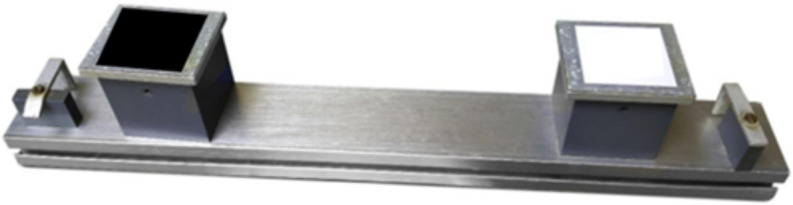
Test apparatus with the two laminated cards (one black, and one white) attached to the metal lids (using magnetic tape) on top of the two PVC boxes

The test apparatus consisted of a metal bar of 50 × 6 cm, with two PVC boxes (5 × 5 × 5 cm) attached to the bar at a distance of 22 cm. Two laminated cards (5 × 5 cm), one white and one black, could be attached to the boxes’ slightly larger metal lids (6 cm x 6.8 cm) using magnetic tape (see Fig. 1). The tight-fitting lids of the apparatus and control trials that were occasionally interspersed between regular trials virtually exclude the possibility that the animals could have used the odor of the food reward (a quarter of dry biscuit) as a cue for their decisions. During these control trials, both containers were baited with a food reward. The performance of the animals in these control trials did not differ from their performance in regular trials. Further, the dry biscuits were near odorless and the animals never tried to sniff at the containers which were in any case at distance of at least 20 cm from their nose.

### Behavioral test

The test apparatus represented a two-alternative choice paradigm in which the animals were allowed to choose between and open one of the two boxes. The box fitted with the laminated card assigned as the rewarded stimulus (S+, e.g. the white card) contained a food reward (a quarter of a dry biscuit) whereas the box fitted with the laminated card assigned as the non-rewarded stimulus (S-, e.g. the black card) was empty. In the first part of the study, we assessed whether or how quickly the spider monkeys learn the association between the visual stimulus assigned as S + and the food reward. In the second part of the study, we assessed whether or how quickly the spider monkeys learn that the reward value of the two visual stimuli has changed, with the rewarded stimulus (S+) now being the non-rewarded one (S-), and vice versa. Subsequently, we assessed with how many of such stimulus reversals the animals succeed within a pre-set number of sessions and if they show an increase in their learning speed across reversals.

### Procedure

Each session started with the experimenter calling an animal to place itself on a perch at the mesh. The experimenter then fitted one of the boxes of the test apparatus with the laminated card assigned as the rewarded stimulus (S+) and placed a food reward inside this box. The other box was fitted with the laminated card assigned as the unrewarded stimulus (S-) and was kept empty. This was done with the experimenter’s back to the animal so that the latter could not see where the food reward was placed. The experimenter then turned around and, now facing the animal, approached the mesh and presented the apparatus a few centimeters away from and parallel to the mesh. The animal looked at both boxes and then reached through the mesh and selected one of them by opening its lid. Opening the box bearing the laminated card assigned as S + allowed the animal to retrieve the food reward whereas opening the box bearing the S- allowed the animal to discover that the box was empty. The experimenter then stepped back so that the apparatus was out of reach for the animal, turned around, recorded the animal’s decision, and prepared the apparatus for the next trial. Although not systematically recorded, there was not a single instance of an observer and the experimenter diverging in their coding of an animal’s decisions.

Each session comprised 10 trials and the placement of the laminated card assigned as the S + was pseudo-randomized, taking care to present the S + equally often on the left and on the right box, respectively. This was done to minimize the risk of animals developing a side bias. The end of each session was indicated to the animal by presenting it with a piece of a dry biscuit.

Six of the animals were assigned to learn that the laminated card bearing the white square was the S+, and the other six animals were assigned to learn that the black square was the S+. One to two sessions were performed per day and animal, depending on the animal’s willingness to cooperate.

Once an animal reached the learning criterion in the initial learning task (e.g. white square assigned as S+, and black square assigned as S-), the reward contingencies of the two stimuli were reversed so that the animal was now presented with the black square as S + and the white square as S-. When the animal reached the learning criterion in this first reversal task, the reward contingencies were reversed again so that the white square was now again assigned as S + and the black square as S-. In this way, a total of 80 sessions, i.e. 800 trials, was performed with each animal.

To minimize the risk of observational learning affecting our results, care was taken that animals tested in neighboring compartments of their enclosure were assigned different colors as their respective S+. The opportunity to interact with their conspecifics when not being tested was clearly more attractive for the animals compared to the opportunity to observe the animal that was tested at a given point in time in a neighboring compartment. Accordingly, although we cannot completely rule out the theoretical possibility of observational learning, we have no reason to assume that this affected our results.

### Data analysis

An animal was considered to have reached the learning criterion when it scored 80% correct responses over two consecutive sessions (each consisting of ten trials) twice in a row (two-tailed binomial test, *p* < 0.05). Thus, an animal had to score at least 16 correct responses out of 20 trials when the data from the first and the second session were pooled, and then again when the data from the second and third session were pooled. This was done to ensure robust learning of the task. The minimal number of sessions to reach this learning criterion was therefore three sessions.

We used the Wilcoxon signed-rank test to assess whether the number of sessions needed for reaching the learning criterion in the initial learning task and in the first reversal learning task, respectively, differed from each other. We used the Mann-Whitney U-test to assess whether groups (e.g. males and females, respectively, or animals assigned the white square or the black square, respectively, as S+) differed from each other in a given task. We used the Kendall’s tau rank correlation test to assess whether the number of sessions needed for reaching the learning criterion across consecutive reversal tasks exhibited a systematic decrease.

## Results

### Initial learning task

**Fig. 2:**
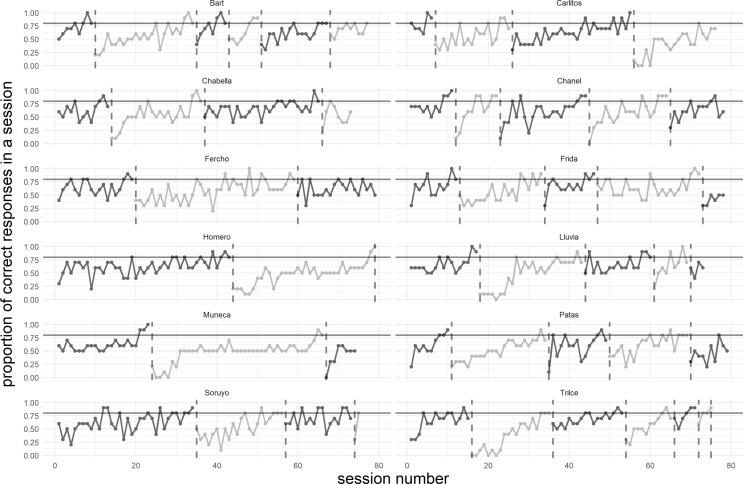
Performance of 12 spider monkeys during the serial visual reversal learning task. Each data point represents one session of 10 trials. A new line in a different shade of grey appears after each reversal, and a dotted vertical line indicates when an animal reached the learning criterion

All 12 animals succeeded with reaching the learning criterion in the initial learning task (Fig. 2). The number of sessions that the animals needed to reach the learning criterion in this task ranged between 6 sessions for the fastest-learning animal and 43 sessions for the slowest-learning one. (Please note that the minimum number of sessions to reach the learning criterion was 3.) As a group, the spider monkeys needed 17.7 (± 10.9) sessions to reach the learning criterion in this task.

Animals that had been assigned the laminated card bearing a white square as the S + did not differ significantly in their mean number of sessions needed to reach the learning criterion (20.7 ± 13.4 sessions) from animals that had been assigned the black square as S+ (13.4 ± 4.4 sessions) (Mann-Whitney U-test, *p* > 0.05). Similarly, males and females did not differ significantly from each other in their learning speed in the initial learning task (males: 18.9 ± 14.2 sessions; females: 16.0 ± 4.4 sessions; Mann-Whitney U-test, *p* > 0.05).

### First reversal learning task

All 12 animals succeeded with reaching the learning criterion in the first reversal learning task (Fig. 2). The number of sessions that the animals needed to reach the learning criterion in this task ranged between 11 sessions for the fastest-learning animal and 43 sessions for the slowest-learning one. (Please note that here, too, the minimum number of sessions to reach the learning criterion was 3.) As a group, the spider monkeys needed 25.8 (± 9.3) sessions to reach the learning criterion in this task. Animals that had now been assigned the laminated card bearing a white square as the S + did not differ significantly in their mean number of sessions needed to reach the learning criterion (27.2 ± 7.4 sessions) from animals that had now been assigned the black square as S+ (24.9 ± 11.0 sessions) (Mann-Whitney U-test, *p* > 0.05). And here, too, males and females did not differ significantly from each other in their learning speed in the first reversal learning task (males: 25.3 ± 9.9 sessions; females: 26.6 ± 9.4 sessions; Mann-Whitney U-test, *p* > 0.05).

10 out of 12 animals needed more sessions to reach the learning criterion in this first reversal learning task compared to the initial learning task. Accordingly, as a group, the animals needed significantly more sessions to reach the learning criterion in the first reversal learning task (25.8 ± 9.3 sessions) than in the initial learning task (17.7 ± 10.9 sessions) (Wilcoxon, *p* < 0.05).

### Serial reversal learning-set formation

**Fig. 3:**
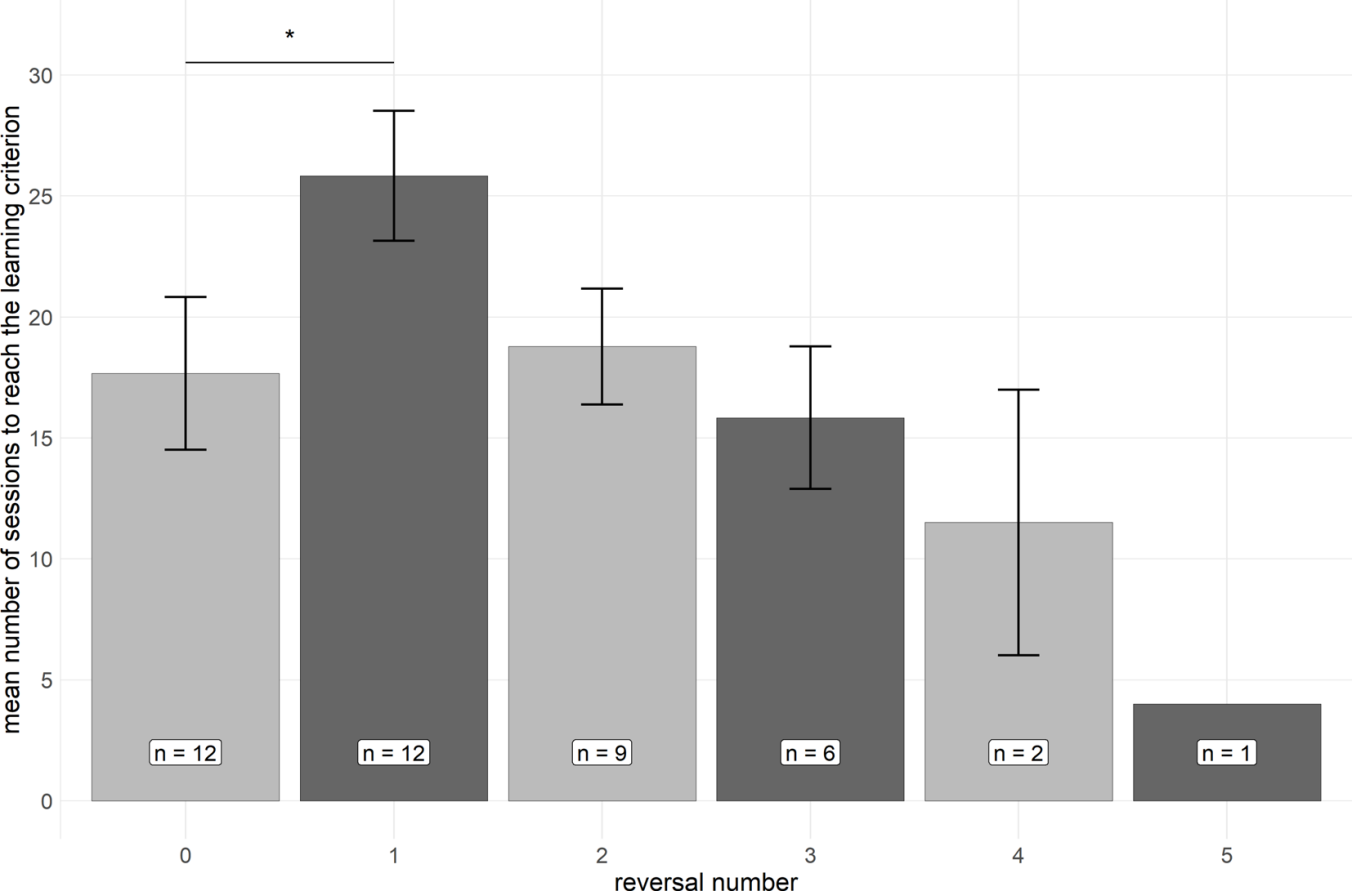
Mean (± SE) number of sessions to reach the learning criterion during the initial learning and during each reversal. Please note that the number of animals decreases from *n* = 12 to *n* = 1 across reversals. The asterisk indicates a significant difference between the two means (Wilcoxon, *p* < 0.05)

Within the 80 sessions performed with all animals, 9 out of 12 animals succeeded with reaching the learning criterion in a second stimulus reversal, 6 animals with a third one, 2 animals with a fourth, and 1 animal even with a fifth stimulus reversal (Fig. 3). There was a significant decrease in the number of sessions needed for reaching the learning criterion across reversals (Kendall’s tau, τ = -0.47, *p* < 0.01).

## Discussion

The results of the present study demonstrate that spider monkeys quickly learned to reliably discriminate between two simultaneously presented visual stimuli, that they succeeded in a visual reversal learning task, and that they displayed an increase in learning speed across consecutive reversals.

### Initial learning task

Our finding that all 12 spider monkeys succeeded with learning to reliably discriminate between two simultaneously presented visual stimuli should not be surprising as *Ateles geoffroyi* had previously been reported to succeed in a visual discrimination task with three-dimensional objects (Shell and Riopelle 1958). Although we employed an unusually tough learning criterion in order to ensure robust learning of the reward values of the two stimuli (see Methods section), our animals needed, on average, only 177 trials to be considered as mastering the initial visual discrimination task. Nonprimate mammal species such as rats (Kumar et al. 2015), mice (van den Broeck et al. 2019), and harbor seals (Erdsack et al. 2022) have been reported to need an average of 640, 400, and 500 trials, respectively, to reach criterion in corresponding visual discrimination learning tasks. Thus, the learning speed of our spider monkeys was clearly higher compared to these nonprimate mammal species. Common marmosets (*Callithrix jacchus*) and rhesus macaques (*Macaca mulatta*) needed, on average, 180 and 200 trials, respectively, to reach learning criterion in a visual discrimination task and thus a similar number compared to the spider monkeys of the present study (Roberts et al. 1988; Butler 1953). Interestingly, chimpanzees (*Pan troglodytes*) have been reported to need, on average, 300 and 1260 trials, respectively, and thus a markedly higher number of trials compared to our spider monkeys to reach criterion in a visual discrimination task (Deaux et al. 2021; Tomonaga and Ohta 1990).

### First reversal learning task

Our finding that all 12 spider monkeys succeeded with reaching the learning criterion in the first reversal learning task is not trivial as several studies on visual reversal learning in both primates and nonprimate mammal species reported that not all, but only some individuals succeeded with a first reversal (e.g. rats: Kumar et al. 2015; mice: van den Broeck et al. 2019; harbor seals: Erdsack et al. 2022; chimpanzees: Cantwell et al. 2022).

We found that the spider monkeys needed, on average, 258 trials to reach the learning criterion in the first reversal and thus significantly more trials compared to the initial learning in which the animals needed an average of 177 trials (see Fig. 3). This is in line with virtually all studies on visual reversal learning that adopted a learning criterion (rather than a fixed number of trials per problem paradigm) in both nonprimate mammals (e.g. rats, Kumar et al. 2015; mice: Laughlin et al. 2011) and nonhuman primates (e.g. common marmosets, Roberts et al. 1988; chimpanzees, Cantwell et al. 2022).

A widely used and intuitive measure to quantify the phenomenon that animals commonly need more trials to reach learning criterion in the first reversal condition than in the initial learning of a visual discrimination task is the Reversal Index (RI). It is defined as the ratio between the number of trials (or errors) to criterion on the first reversal learning divided by the number of trials (or errors) to criterion on the initial discrimination learning (Rajalakshmi and Jeeves 1965). If, for example, an animal needs 250 trials to reach criterion during the reversal condition, and 100 trials during the initial learning, then the Reversal Index is 250 divided by 100 = 2.5.

As stimulus reversal learning is considered to be a measure of behavioral flexibility and inhibitory control (Shettleworth 2010; Lea et al. 2020), the Reversal Index allows for between-species comparisons of these cognitive skills. Table 1 summarizes the RI values of several primates, nonprimate mammals, and birds.

**Table 1:** Reversal index of primates, nonprimate mammals, and birds tested on visual reversal learning using a learning criterion approach

Common name	Scientific name	Reversal Index	Ref.
***Primates***			
Human subjects	*Homo sapiens*	0.32	[1]
Rhesus macaque	*Macaca mulatta*	1.33	[1]
Spider monkey	*Ateles geoffroyi*	1.47	[x]
Long-tailed macaque	*Macaca fascicularis*	1.62	[10]
Chimpanzee	*Pan troglodytes*	1.78	[15]
Grey mouse lemur	*Microcebus murinus*	1.86	[14]
Stump-tailed macaque	*Macaca arctoides*	1.90	[13]
Common marmoset	*Callithrix jacchus*	2.30	[12]
Squirrel monkey	*Saimiri sciureus*	2.83	[2]
Capuchin monkey	*Sapajus apella*	2.86	[11]
Tonkean macaque	*Macaca tonkeana*	2.95	[10]
Lion-tailed macaque	*Macaca silenus*	2.99	[11]
***Nonprimate mammals***			
Coatimundi	*Nasua narica*	1.25	[2]
Cat	*Felis catus*	1.82	[1]
Skunk	*Mephitis mephitis*	1.94	[2]
Grey kangaroo	*Macropus giganteus*	1.96	[4]
Dog	*Canis lupus familiaris*	2.20	[6]
Pig	*Sus scrofa domesticus*	2.33	[5]
Harbor seal	*Phoca vitulina*	2.34	[9]
Mouse	*Mus musculus*	2.50	[7]
Brown rat	*Rattus norvegicus*	2.55	[3]
Horse	*Equus ferus caballus*	2.86	[8]
***Birds***			
Red-billed blue magpie	*Urocissa occipitalis*	1.04	[2]
Pigeon	*Columba livia*	1.78	[2]
Greater hill myna	*Gracula religiosa*	2.12	[2]
Ringneck dove	*Streptopelia risoria*	2.68	[2]
Bob-white quail	*Colinus virginianus*	2.75	[2]
Trumpeter	*Psophia crepitans*	3.08	[2]
Chukar	*Alectoris chukar*	3.32	[2]
White-leghorn chicken	*Gallus gallus domesticus*	4.16	[2]
Common crow	*Corvus brachyrhynchos*	6.25	[2]

[x] present study; [1] Rajalakshmi and Jeeves (1965); [2] Gossette and Gossette (1967); [3] Jeeves (1967); [4] Munn (1964); [5] Moustgaard et al. (2004); [6] Tapp et al. (2003); [7] Glynn et al. (2016); [8] Sappington et al. (1997); [9] Erdsack et al. (2022); [10] Loyant et al. (2023); [11] Judge et al. (2011); [12] Jackson et al. (2019); [13] Anderson et al. (1996); [14] Joly et al. (2014); [15] Cantwell et al. (2022)

Humans are the only species so far who needed fewer trials in the reversal condition than in the initial learning and thus score an RI value < 1. Although primates, on average, score a lower mean RI value (2.01 ± 0.81) compared to nonprimate mammals (2.18 ± 0.45) and birds (3.02 ± 1.51), respectively, there is considerable overlap in RI values between these taxa and, accordingly, they do not differ significantly from each other when compared pairwise (primates vs. nonprimate mammals: z=-0.53, *p* = 0.59; primates vs. birds: z=-1.56, *p* = 0.12; nonprimate mammals vs. birds: z=-1.43, *p* = 0.15; Mann-Whitney U-test).

The spider monkeys of the present study scored the second-lowest RI value among the nonhuman primates (1.47), only surpassed by rhesus macaques (1.33), and thus even outperformed chimpanzees (1.78) in this measure of cognitive performance. (Please note that the RI value reported here for the spider monkeys is based on the performance of 15 animals: the 12 animals that performed the complete study with 800 trials each, plus three animals which only performed the initial learning and the first reversal task and then escaped from the field station.) This raises the question as to possible reasons which may underlie the observed variation among primates (and other taxa) with regard to this cognitive skill.

Differences in relative brain size, quantified as the Encephalization Quotient (EQ), have repeatedly been put forward to explain between-species differences in cognitive skills (e.g. Williams 2002; Roth and Dicke 2005). However, recent studies suggest that the EQ may be an inadequate measure to predict cognitive performance (van Schaik et al. 2021). This notion is also supported by the fact that the EQ values reported in the literature (Williams 2002) do not significantly correlate with the RI values of the primates listed in Table 1 (Spearman, r_s_=-0.28, *p* > 0.05).

Other studies suggest that absolute brain size rather than relative brain size would be predictive of cognitive performance in primates (e.g. Deaner et al. 2007). However, it is unclear whether such a correlation, if it exists, may only hold true when comparing measures of general cognitive ability between species such as the *g* factor, or whether absolute brain size may also predict task-specific cognitive abilities (Amici et al. 2010; Schmitt et al. 2012).

Yet other studies propose that the relative size of certain brain structures rather than the brain’s relative or absolute size may determine a species’ performance with regard to a given cognitive skill (Dunbar and Shultz 2017). Stimulus reversal learning is one of the few cognitive tests for which neurophysiological studies succeeded in defining the brain areas that are involved. Lesion studies and functional neuroimaging in human and nonhuman primates demonstrated that the orbitofrontal cortex and the medial prefrontal cortex seem to be critically involved in this type of learning (Clark et al. 2004; Rygula et al. 2010; Izquierdo et al. 2017). Unfortunately, exact values for the volume of these brain areas in those nonhuman primate species which have been assessed for their performance in stimulus reversal tasks are, to the best of our knowledge, not at hand. Similarly, it would be interesting to assess whether the large variation in RI values found among nonprimate mammals and among birds (Table 1) – with some species such as the coatimundi and the red-billed blue magpie scoring even lower values than the best nonhuman primates – might be explained by between-species differences in neuroanatomical correlates of cognition and/or by differences in socio-ecological factors.

Recent research suggests that socio-ecological factors such as dietary specialization and social complexity rather than neuroanatomical traits may better explain between-species differences in cognitive performance (Grabowski et al. 2023). Accumulating evidence supports the notion that frugivory, for example, may be a driver of cognition in primates as the complex spatial and temporal distribution patterns of fruit require cognitive skills such as the ability to form and use mental maps of the foraging area, to build and retrieve long-term spatial and sensory memory for successful foraging and food selection, and decision-making competence that are needed to a lesser degree with other dietary specializations such as folivory (DeCasien et al. 2017).

Similarly, accumulating evidence supports the notion that social complexity may be a driver of cognition in primates as the need to keep track of changes in social relationships, the ability to recognize and to communicate with group members, as well as fission-fusion dynamics are thought to require enhanced cognitive skills (Peckre et al. 2019; Shultz and Dunbar 2022). In line with this notion, a recent study reported that a socially tolerant primate species displayed more inhibitory control and thus better performance in reversal learning compared to two socially intolerant primate species (Loyant et al. 2023). Similarly, another study reported that primate species with low inhibitory control, measured as the frequency of self-directed behavior, scored poorer on reversal learning than species with high inhibitory control (Judge et al. 2011).

The fact that spider monkeys are highly frugivorous (Gonzalez-Zamora et al. 2009), live in multi-male/multi-female groups, and display strong fission-fusion dynamics (Campbell 2008) is consistent with the idea that socio-ecological factors such as dietary specialization and social complexity may explain differences in cognitive abilities as the spider monkeys of the present study scored the second-lowest RI value among all nonhuman primate species tested so far on visual reversal learning, indicative of a high degree of behavioral flexibility and inhibitory control. Exactly these two traits should be particularly beneficial for spider monkeys both with regard to decision-making in the context of foraging and food selection (e.g. deciding for a certain fruit-bearing tree or for a fruit with a certain degree of ripeness) as well as in the context of social interactions (e.g. deciding for joining a certain foraging party or for tracking the ever-changing social relationships within their troop).

Capuchin monkeys have repeatedly been reported to score relatively high in tasks of both physical and social cognition (Woodley of Menie and Peñaherrera-Aguirre 2023). It is therefore somewhat surprising that they scored a markedly higher RI value, and thus a lower cognitive performance, compared to the spider monkeys of the present study (Table 1). However, in contrast to spider monkeys, capuchins show low levels of fission-fusion dynamics (Amici et al. 2008) and are less frugivorous than spider monkeys (Hawes and Peres 2014). Future studies should therefore aim to elucidate if one, or perhaps both of these socioecological traits may explain the marked difference between these two primate species in visual reversal learning.

### Serial reversal learning-set formation

Our finding that the spider monkeys displayed a significant decrease in the number of sessions needed for reaching the learning criterion across reversals (see Fig. 3) and thus a clear increase in their learning speed across consecutive reversals suggests that *Ateles geoffroyi* is capable of serial reversal learning-set formation with visual cues. The average number of sessions needed for reaching the learning criterion decreased from 25.8 (± 9.3) sessions for the first reversal over 18.8 (± 7.2), 16 (± 7.1), and 11.5 (± 7.8) sessions in the second, third, and fourth reversal, respectively, to only 4 sessions in the fifth reversal. Accordingly, the animals needed less than half the number of sessions to reach criterion in the fourth reversal compared to the first reversal (Fig. 3).

Learning-set formation is defined as “learning how to learn efficiently in situations that an animal encounters frequently” (Harlow 1949). Most commonly, learning-set formation is assessed using a succession of *different* stimulus pairs (each pair with one stimulus assigned as S + and one assigned as S-) and the ability of an animal to need fewer and fewer trials to learn the reward value of each stimulus across successive stimulus pairs (or an increase in trial-2 performance across stimulus pairs) is considered as evidence of the ability to build an abstract learning rule or concept (Shettleworth 2010). Much fewer studies so far assessed whether animals are also able to form a learning-set when the reward value of the two stimuli in the *same* stimulus pair is repeatedly switched as is the case in serial reversal learning.

Using a fixed number of trials per problem approach rather than a performance criterion approach, nonprimate mammals such as rats (Mackintosh et al. 1985 and cats (Warren 1966), as well as primate species such as rhesus macaques (Harlow 1949), squirrel monkeys and capuchins (Gossette and Inman 1966), common marmosets (Cotterman et al. 1956), and chimpanzees (Schusterman 1962) showed at least some degree of increase in trial-2 performance across problems which can be considered as learning-set formation within a serial visual reversal task. The only study on serial visual reversal learning-set formation which employed a performance criterion approach – similar to the approach adopted in the present study – rather than a fixed number of trials per problem approach found that one out of four harbor seals showed progressively improving performance across reversals (Erdsack et al. 2022).

Considering the high performance of spider monkeys in other tests of physical cognition which in several cases matches or even surpasses the performance of great apes (Deaner et al. 2006; Reader et al. 2011; Bosshard et al. 2022), it may seem surprising that the spider monkeys of the present study failed to reach one-trial learning in serial visual reversal learning. However, to the best of our knowledge, all studies that reported one-trial learning in this type of task employed a fixed number of trials per problem approach instead of a performance criterion approach (e.g. chimpanzees: Schusterman 1964; rats: Mackintosh et al. 1968). Further, as we employed a performance criterion approach, we were only able to perform a limited number of stimulus reversals – five instead of the hundreds of reversals that are typically performed with the fixed number of trials approach, and which are needed by rats and chimpanzees to achieve one-trial learning (Schusterman 1964; Mackintosh et al. 1968). Thus, we cannot exclude the possibility that at least some of our animals may have achieved or approached one-trial learning if time would have allowed for a sufficiently high number of reversals to be performed.

In summary, we found that captive spider monkeys quickly learned to reliably discriminate between two simultaneously presented visual stimuli, that they succeeded in a visual reversal learning task, and that they displayed an increase in learning speed across consecutive reversals. They outperformed most other primate species tested so far on serial visual reversal learning, including chimpanzees, with regard to their learning speed in both the initial learning task and in the first reversal task. Our findings support the notion that socio-ecological factors such as dietary specialization and social complexity foster advanced cognitive skills.

## Data Availability

All relevant data are presented in the manuscript. Detailed data are available from the corresponding author upon reasonable request.
